# Design of an HF-Band RFID System with Multiple Readers and Passive Tags for Indoor Mobile Robot Self-Localization

**DOI:** 10.3390/s16081200

**Published:** 2016-07-29

**Authors:** Jian Mi, Yasutake Takahashi

**Affiliations:** Department of Human and Artificial Intelligent Systems, Graduate School of Engineering, University of Fukui, 3-9-1, Bunkyo, Fukui 910-8507, Japan; yasutake@ir.his.u-fukui.ac.jp

**Keywords:** HF-band RFID, self-localization, novel likelihood function, low production cost

## Abstract

Radio frequency identification (RFID) technology has already been explored for efficient self-localization of indoor mobile robots. A mobile robot equipped with RFID readers detects passive RFID tags installed on the floor in order to locate itself. The Monte-Carlo localization (MCL) method enables the localization of a mobile robot equipped with an RFID system with reasonable accuracy, sufficient robustness and low computational cost. The arrangements of RFID readers and tags and the size of antennas are important design parameters for realizing accurate and robust self-localization using a low-cost RFID system. The design of a likelihood model of RFID tag detection is also crucial for the accurate self-localization. This paper presents a novel design and arrangement of RFID readers and tags for indoor mobile robot self-localization. First, by considering small-sized and large-sized antennas of an RFID reader, we show how the design of the likelihood model affects the accuracy of self-localization. We also design a novel likelihood model by taking into consideration the characteristics of the communication range of an RFID system with a large antenna. Second, we propose a novel arrangement of RFID tags with eight RFID readers, which results in the RFID system configuration requiring much fewer readers and tags while retaining reasonable accuracy of self-localization. We verify the performances of MCL-based self-localization realized using the high-frequency (HF)-band RFID system with eight RFID readers and a lower density of RFID tags installed on the floor based on MCL in simulated and real environments. The results of simulations and real environment experiments demonstrate that our proposed low-cost HF-band RFID system realizes accurate and robust self-localization of an indoor mobile robot.

## 1. Introduction

Most of the developed countries are facing the problems of the aging of the human population and labor shortage. Then, intelligent mobile service robots have been developed with the main purpose of solving these problems. Consequently, one of the tasks that needs to be accomplished on an urgent basis is highly accurate and robust self-localization of robots. Many types of research have been conducted on the self-localization of indoor mobile robots. Several of the systems developed for this purpose employ vision sensors [1,2], lasers [3], ultrasonic sensors [4], infrared sensors [5], radar [6] and ultrasonic technology [7]. However, these conventional approaches are not robust or accurate enough to localize an indoor mobile robot, given the presence of several kinds of disturbances. Vision sensors suffer from the change of illumination and environment conditions. Laser range finders (LRFs) cannot locate a robot accurately if the robot is surrounded by many unknown moving obstacles or when transparent walls are widely used in the environment, because LRF fails to detect them. Further, the performance of ultrasonic sensors is easily affected by obstacles around the robot.

**Figure 1:**
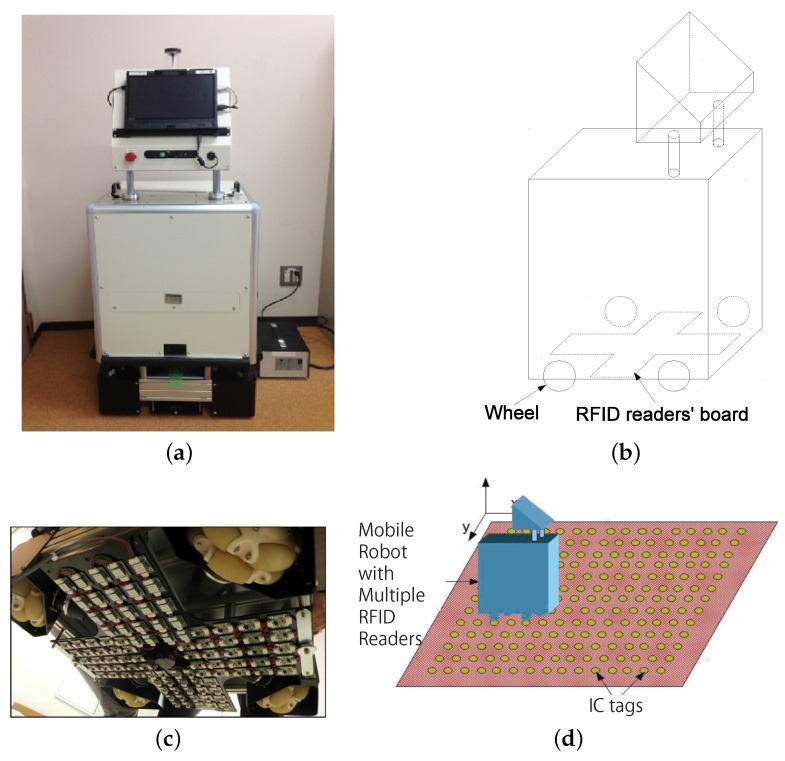
Self-localization of an indoor mobile robot using multiple RFID readers and RFID tags. (**a**) Omni directional vehicle; (**b**) structure; (**c**) multiple readers; (**d**) self-localization model.

A radio frequency identification (RFID) system has recently attracted attention as one of the alternative sensing devices. RFID technology employs electromagnetic fields to transfer data between an RFID tag and an RFID reader. Each RFID tag has its own unique ID information, and RFID tags are inexpensive enough to be distributed in large densities among objects and environments. RFID systems are widely used for applications, such as identification, transpiration tracking and inventory control. These systems help to realize relatively robust wireless communication to exchange data between a tag and a reader. Researchers in the field of robotics have investigated the use of an RFID system for self-localization of a mobile robot. This can be achieved by two approaches. One is to use the RFID system to compensate for another self-localization system based on a vision sensor, LRF or GPS for instability or a huge exploration space [8,9,10]. However, the disadvantages of the base self-localization system remain in this approach. The other approach is to use only an RFID system for self-localization [11,12,13,14,15,16]. Various methods for making use of an RFID system for self-localization have been proposed. One method is to use only kinematic constraints [13,14,15]. However, the accuracy of self-localization depends on the density of tags; therefore, a high density of tags is required for accurate self-localization. Another method is based on a machine learning approach [11]; however, the RFID system is adversely affected by obstacles surrounding the reader and tags. Yet another method entails the adaptation of probabilistic approaches [12,16]. In particular, the Monte-Carlo localization (MCL) method [17] enables self-localization with relative accuracy. A previous study [12] employed relatively large-tags and small readers. However, the large size of tags resulted in a large localization error. Another study [16] employed small tags and a large number of readers with small antennas. Figure 1 shows an omni-directional vehicle equipped with an HF-band RFID system, as employed in the previous study [16]. In this RFID system, 96 RFID readers are installed at the bottom of the robot. A high density of RFID tags is embedded in carpets on the floor. The RFID self-localization system with 96 readers is quite robust, and it works flawlessly even in the case of stains or marker tapes on the carpet. However, the production cost of this RFID system with 96 RFID readers is relatively high. This drawback prevents widespread utilization of an RFID-based self-localization system in service robots employed in public facilities, such as those serving in hospitals. There is a high demand for a low-cost RFID system. Furthermore, although MCL enables robust self-localization, the estimated initial position and posture are crucial for rapid recovery after the robot has lost its position and posture.

This paper proposes a novel configuration of a high frequency (HF)-band RFID system that employs fewer RFID readers, has a low density of passive tags and a low production cost and that maintains the self-localization accuracy. We find through experiments with real robots that the measurement model of the RFID system cannot be modeled by using a Gaussian distribution if the antenna is enlarged in size. We show that a likelihood function that considers the antenna size contributes to the accuracy of the self-localization. The key contributions of this study are as follows.

1. A novel particle reinitialization method based on MCL is proposed to enable rapid self-localization.
2. A likelihood function that considers the antenna size is proposed for accurate self-localization.
3. An HF-band RFID system with eight RFID readers placed in a new arrangement is developed by increasing the antenna size of the RFID reader.

Eventually, the proposed RFID system provides highly robust self-localization of an indoor mobile robot at low production cost.

## 2. Related Works

A basic RFID system consists of three components, a transceiver (commonly known as the RFID reader), a transponder (commonly known as the RFID tag) and an antenna. Depending on the communication method, RFID systems can be divided into two types. One type uses radio waves, and the other uses electromagnetic induction for communication between the transceiver and the transponder. Ultra-high-frequency (UHF)-band and super-high-frequency (SHF)-band RFID systems are based on the use of radio waves, which can realize long distance communication. HF-bandand low-frequency (LF)-band RFID systems use electromagnetic induction. The communication distance of HF-band and LF-band RFID systems is shorter than that of a UHF-band or SHF-band RFID system; however, an HF-band or LF-band RFID system is much more stable and accurate for tag detection and more robust against obstacles in the environment and environmental changes. LF-band, HF-band or UHF-band RFID systems have already been utilized for mobile robot self-localization. Details of related works on robot self-localization works are listed in Table 1.

Miguel Pinto et al. [18] used an omnidirectional camera and LRF sensors to realize robot self-localization. Their self-localization system performed well, and the average self-localization was less than 80 mm. However, in general, vision sensors suffer from the problem of illumination changes, and LRF sensors are unable to accurately locate the robot when numerous transparent walls are present in the robot’s environment.

Dirk Hahnel et al. [8] proposed a combination system composed of a UHF-band RFID reader and an LRF. They used a probabilistic measurement model for RFID readers to locate RFID tags. In their system, two RFID reader antennas were installed on the robot, and tags were attached to walls, furniture, etc. The RFID system was used only to compensate for the global positioning of the localization based on the LRF. Then, the accuracy of their system was dependent on the LRF-based self-localization system, which is sensitive to the condition of unexpected obstacles in the robot’s environment. It is preferable to construct a single RFID system without any other sensors, which can be used in a dark environment or in an environment containing several transparent walls, such as in hospitals and other public facilities.

Lei Yang et al. [19] proposed a hybrid particle filter method for object tracking using a UHF-band RFID system. This method is more computationally efficient than the particle filter while providing the same accuracy. The limitation of their system is that it can locate only the position of the robot and cannot estimate its orientation. However, for autonomous navigation of indoor mobile robots, high accuracy localization of the position and orientation of the robot is necessary. The UHF-band RFID system of Lei Yang [19] realized self-localization with an error of about 186 mm, in contrast to the much higher accuracy of localization of the position and orientation achieved in our previous work, where we propose the use of a novel particle reinitialization method based on MCL to enable rapid self-localization. Instead of using the conventional Gaussian function (i.e., Gaussian model) as the likelihood model, we newly design a likelihood model that is a combination of the Gaussian distribution and the step function, which can improve the self-localization accuracy.

Both Park et al. [13] and Han et al. [14] proposed HF-band RFID systems for self-localization. Park et al. [13] used only one RFID reader antenna in their system. However, it is difficult to estimate the orientation of the robot with only one reader antenna. Han et al. [14] proposed a new triangular pattern for arranging the RFID tags on the floor, instead of the conventional square pattern, and they achieved accurate localization with an localization error of about 16 mm. However, their system suffers from the same problem as that of Park et al. [13] because it also uses only one RFID reader. A common approach to solving this problem is to combine these systems with other sensors [20,21]. They developed a new localization method that uses trigonometric functions to estimate the position and orientation with only one RFID reader. Unfortunately, the system they developed cannot ensure the reliability of localization, especially when the robot moves quickly or when no tags are detected. HF-band RFID systems have also been utilized for object pose estimation [22] or communication robots [23]; however, the objectives of these works were different from those of our study.

Yang et al. [24] used one HF-band RFID reader with a large antenna size of 660 × 300 mm${}^{2}$. They derived an exponential-based function to reflect the relationship between RFID tag distribution and localization precision. They proposed an approach of using sparsely-distributed passive RFID tags. They used a simple and efficient localization algorithm proposed by Han et al. [14]. The RFID system with sparse RFID button tag distribution patterns realized better localization with precisions of about 36 mm and 38 mm in the *x* direction and *y* direction, respectively. However, this system was unable to realize real-time localization. The robot stayed for 40 s at each point for localization. In addition, the system could not estimate the orientation of the robot. Furthermore, installation of their sparse RFID tag distribution patterns in an indoor environment was difficult. Yang and Wu [25] proposed a particle filter algorithm using a position-information-based straight observation model and a 2D Gaussian-based motion model to locate the robot. They used a dense tag distribution. Other experimental conditions in their study were the same as those in Yang et al. [24]. They set the localization accuracy as 100 mm and the localization precisions of about 27 mm and 46 mm in the *x* direction and *y* direction, respectively. However, their system suffers from the same problems as did that of Yang et al. [24]. In the present research, we designed a novel HF-band RFID system with eight RFID readers having an appropriately-sized antenna to eliminate uncertainties and to realize real-time position and orientation localization.

Mohd Yazed Ahmad et al. [26] proposed a novel triangular-bridge-loop reader antenna for positioning and presented a method for the improvement of HF-RFID-based positioning. They used an HF-band RFID reader with a large-sized antenna having dimensions of 320 × 230 mm${}^{2}$, which was designed with a novel triangular-bridge-loop. In their system, passive RFID tags were sparsely distributed at a distance of about 1300 mm. Their system performed quite well and achieved an average positioning error of 40.5 mm, in contrast to the average positioning error of 124.1 mm in the case of a system employing a conventional reader antenna. However, their system has a limitation in that the speed of the robot is set to be consistent to ensure successful reading of the tags by the reader. If the robot moves faster, a faster reader and tag are required. If the robot moves slower or if an emergent circumstance occurs where the speed is required to be reduced, the system may not detect tags for a long time, as the distance between two tags is 1300 mm. Under this condition, only encoder data can be used for localization, which could make the localization difficult. In our proposed RFID system, there is no such speed limitation. We validate our proposed RFID system at various speeds and, thus, demonstrate its overall efficiency.

An LF-band RFID system for indoor mobile robot self-localization has been proposed in some studies [12,27]. For example, Kodaka et al. [12] used a vehicle equipped with two RFID readers. In their study, RFID tags were installed on the floor, and the size of one tag was 260 × 260 mm${}^{2}$. The distance between adjacent tags was 300 mm. The MCL method was used for robot self-localization, and the localization error was less than 100 mm and 0.1 rad on average. Unfortunately, despite developing an active self-localization method [28], the researchers faced difficulty in increasing the localization accuracy given the dependence on the size of a tag. The communication range of RFID readers also affects the accuracy of self-localization.

Takahashi et al. [15] proposed an RFID-based self-localization system having eight RFID reader antennas at the bottom of the robot. In this system, the RFID tags were arranged in a lattice pattern, and a simple kinematics was used to localize the robot. The accuracy of self-localization was highly dependent on the density of the RFID tags. With eight RFID reader antennas and small-sized high-density RFID tags (100 tags/m${}^{2}$), the localization error was less than 17 mm and 0.12 rad on average. However, if the reader failed to read the tag, the error became relatively large, and the system faced a problem of latency in scanning the tags given that scanning needs to be performed by one antenna after another. Therefore, the system became unstable at high speeds. Takahashi and Hashiguchi [16] developed a new mobile robot self-localization system using MCL based on the HF-band RFID system. This system included 96 RFID readers, and the density of the RFID tags was 100 tags/m${}^{2}$. They compared the performances of self-localization with an RFID system only and with an RFID system equipped with LRFs and found that in the absence of obstacles, both RFID systems were able to locate the robot accurately. However, in the presence of obstacles around the robot, the system using LRFs could not locate the robot accurately and stably, which demonstrated the efficiency of the RFID system used alone.

The limitation of their system is its high production cost. It is then necessary to redesign the system, reduce the number of RFID readers, use a low density of RFID tags and establish an efficient configuration of the system to cut down its production cost.

## 3. Self-Localization of an Indoor Mobile Robot by Using an RFID System with MCL

**Figure 2:**
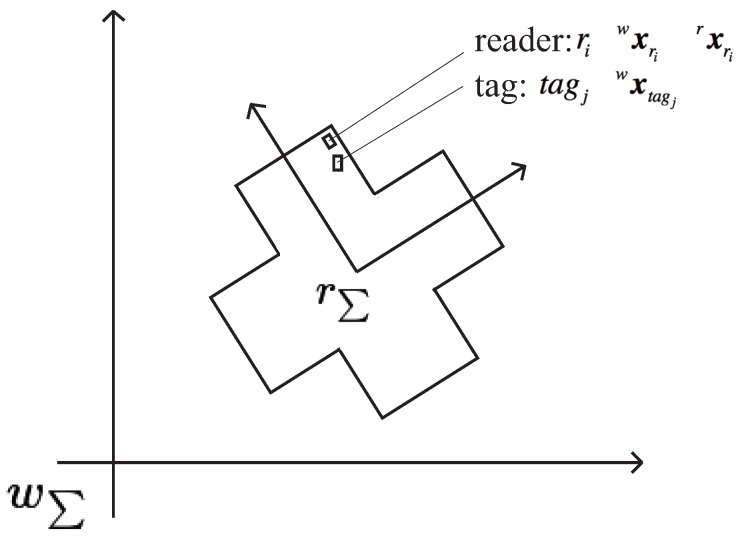
World coordinate system ${}^{w}\Sigma$ and robot coordinate system ${}^{r}\Sigma$.

MCL, one of the probabilistic approaches [17], has been shown to be a good method for real-time self-localization of robots. Takahashi and Hashiguchi [16] applied MCL to their RFID-system-based self-localization. It is assumed that an RFID tag has a unique ID and that the tag ID map is maintained with the position of each tag. RFID readers work independently and asynchronously of each other. We briefly introduce the MCL using the RFID system [16] here. We define the world coordinate system ${}^{w}\Sigma$ and the robot coordinate system ${}^{r}\Sigma$ as shown in Figure 2. The robot position and orientation is defined in the world coordinate system at time *t* as ${}^{w}x_{t}=({}^{w}x_{t},{}^{w}y_{t},{}^{w}\theta_{t})$. $z_{t}=\left(r_{t},tag_{t}\right)$ is the measurement output at time *t*, and $tag_{t}$ is the tag detected with the RFID reader $r_{t}$. A motion model ${}^{w}x_{t+1}=MotionModel({}^{w}x_{t})$ is defined to estimate the next robot position and orientation of the robot, ${}^{w}x_{t+1}$ from ${}^{w}x_{t}$. A measurement model $p(z_{t}|{}^{w}x_{t})$ is also defined to calculate the posterior probability to receive the measurement output $z_{t}$ if the robot position and orientation are ${}^{w}x_{t}$. A set of particles is defined as a set of hypotheses of the robot position and orientation denoted at time *t* as $X_{t}=({}^{w}x_{t}^{\left[1\right]}{,}^{w}x_{t}^{\left[2\right]},\cdots ,{}^{w}x_{t}^{M})$, where *M* is the number of particles. The algorithm of MCL is given in Algorithm 1. The self-localization system updates the particles with a fixed sampling time Δt. If no RFID reader detects a tag within the sampling time, the procedure of belief calculation (Step 4 in Algorithm 1) is skipped.

The motion model of an omnidirectional vehicle is given by Equation (1):

(1) $$\begin{array}{ccc} {}^{w}x_{t} & = & {}^{w}x_{t-1}+V\Delta t+\epsilon \Delta t,\;\epsilon ∼N\left(0,\sigma \right) \end{array}$$

where $V=(v_{x},v_{y},\omega )$, Δt and N(0,σ) denote the velocity of the robot, sampling time and Gaussian distribution with the standard deviation $\sigma =(\sigma_{x},\sigma_{y},\sigma_{\omega })$, respectively.

The position of $tag_{j}$ detected by RFID reader antenna $r_{i}$ in world coordinates is ${}^{w}x_{tag_{j}}=\;{{(}^{w}x_{tag_{j}}{,}^{w}y_{tag_{j}}{,}^{w}z_{tag_{j}})}^{T}$. The position of RFID reader $r_{i}$ at time *t* in world coordinates ${}^{w}x_{r_{i}}=({{}^{w}x_{r_{i}},{}^{w}y_{r_{i}},{}^{w}z_{r_{i}})}^{T}$ is estimated by Equation (2):

(2) $$\left(\begin{array}{c} {}^{w}x_{r_{i}}\\ {}^{w}y_{r_{i}} \end{array}\;\;\;\right)=\left(\begin{array}{cc} \cos{}^{w}\theta_{t} & -\sin{}^{w}\theta_{t}\\ \sin{}^{w}\theta_{t} & \cos{}^{w}\theta_{t} \end{array}\;\;\;\right)\left(\begin{array}{c} {}^{r}x_{r_{i}}\\ {}^{r}y_{r_{i}} \end{array}\;\;\;\right)+\left(\begin{array}{c} {}^{w}x_{t}\\ {}^{w}y_{t} \end{array}\;\;\;\right)$$

where ${\left({}^{r}x_{r_{i}},{}^{r}y_{r_{i}}\right)}^{T}$ is the position of the RFID reader antenna $r_{i}$ in the robot coordinate system and it is known in advance. We assume ${}^{w}z_{tag_{j}}$ and ${}^{w}z_{r_{i}}$ to be constant.

**Algorithm 1** Monte Carlo localization.1:Initialize particles ${\bar{X}}_{t}=X_{t}={(}^{w}x_{t}^{\left[1\right]},{}^{w}x_{t}^{\left[2\right]},\cdots ,{}^{w}x_{t}^{M})$2:**for**
m=1 to *M*
**do**3:  Update particles with the motion model: ${}^{w}x_{t}^{m}=Motionmodel\left({}^{w}x_{t-1}^{m}\right)$  4:  Calculate the belief of each particle with the measurement model: $w^{m}=p\left(z_{t}|{}^{w}x_{t}^{m}\right)$5:  ${\bar{X}}_{t}={\bar{X}}_{t}+<{}^{w}x_{t}^{m},w_{t}^{m}>$6:**end**
**for**7:**for**
m=1 to *M*
**do**8:  draw ${}^{w}x_{t}^{m}$ from ${\bar{X}}_{t}$ with probability ∝ $w_{t}^{m}$9:  add ${}^{w}x_{t}^{m}$ to $X_{t}$10:**end**
**for**11:**for**
m=1 to *M*
**do**12:  if $w_{t}^{m}<\alpha$(a constant), re-initialize ${}^{w}x_{t}$13:**end**
**for**14:return $X_{t}$

Then, the weight of each particle, $w^{m}$, is calculated using the measurement model $p(z_{t}|{}^{w}x_{t}^{m})$. $p(z_{t}{|}^{w}x_{t}^{m})$ is a likelihood function defined in Section 5. After the weights $w^{m}$ are calculated, the algorithm estimates the position of the robot as the weighted mean of the particles.

(3) $${}^{w}x_{t}=\frac{\sum_{m=1}^{M}{}^{w}x_{t}^{m}w^{m}}{\sum_{m=1}^{M}w^{m}}$$

As shown in Steps 7–10 of Algorithm 1, particles are updated with a probability proportional to the weight $w^{m}$.

### Particle Reinitialization

**Figure 3:**
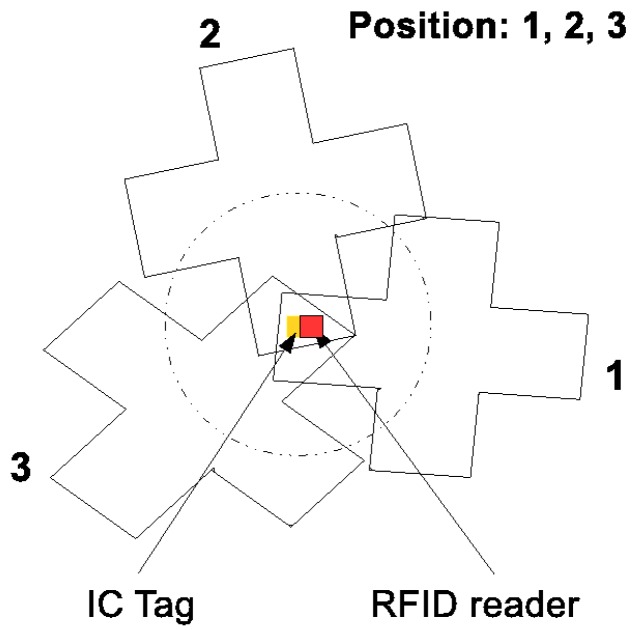
Possible poses of the robot when one reader detects a tag: Positions 1, 2 and 3 are possible poses of the robot when it detects the tag by means of its reader.

In conventional studies, in scenarios where the initial position of the robot was unknown or the weights of all particles became too small during the transportation because of an unexpected disturbance in the robot’s movement, the particles were distributed uniformly randomly in the possible exploration space. However, it is obviously undesirable to distribute the particles uniformly if the possible exploration space is too large. Once the robot detects one of the tags, it can narrow down its own possible position immediately according to the position of the detected tag and the reader that detects it. Figure 3 shows examples of the possible poses of the robot when it detects a tag if its own position is unknown.

We propose a novel particle reinitialization method that is specific to the RFID system and that is aimed at the realization of highly efficient and accurate self-localization. The particle reinitialization process is performed from Step 11 onward in Algorithm 1. Once $w^{m}$ becomes too small for the robot’s self-localization, particles ${}^{w}x_{t}={(}^{w}x_{t}{,}^{w}y_{t}{,}^{w}\theta_{t})$ will be reinitialized as given in Equation (4):

(4) $$\left(\begin{array}{c} {}^{w}x_{t}\\ {}^{w}y_{t} \end{array}\;\;\;\right)=\left(\begin{array}{c} {}^{w}x_{tag_{j}}\\ {}^{w}y_{tag_{j}} \end{array}\;\;\;\right)-\left(\begin{array}{cc} \cos{}^{w}\theta_{t} & -\sin{}^{w}\theta_{t}\\ \sin{}^{w}\theta_{t} & \cos{}^{w}\theta_{t} \end{array}\;\;\;\right)\left(\begin{array}{c} {}^{r}x_{r_{i}}\\ {}^{r}y_{r_{i}} \end{array}\;\;\;\right)$$

where ${}^{w}\theta_{t}$ is generated using a uniform random function from −π to *π*. The re-sampling indicates that the robot is at a position where the RFID reader $r_{i}$ is just above the detected tag $tag_{j}$. The proposed re-sampling leads the self-localization system to localize the robot itself quickly and stably because it can eliminate unnecessary particles distributed over the possible exploration space. In our research, the number of particles is set as 500.

## 4. HF-Band RFID Systems

In this study, we use multiple HF-band RFID readers to realize highly accurate and stable real-time localization. Generally, an RFID reader with a large antenna could provide a large area for tag detection. However, using a large antenna would also increase uncertainty during the localization. To eliminate uncertainty and realize highly accurate real-time localization, we first used 96 HF-band RFID readers with a small antenna.

### 4.1. System with 96 HF-Band RFID Readers

**Figure 4:**
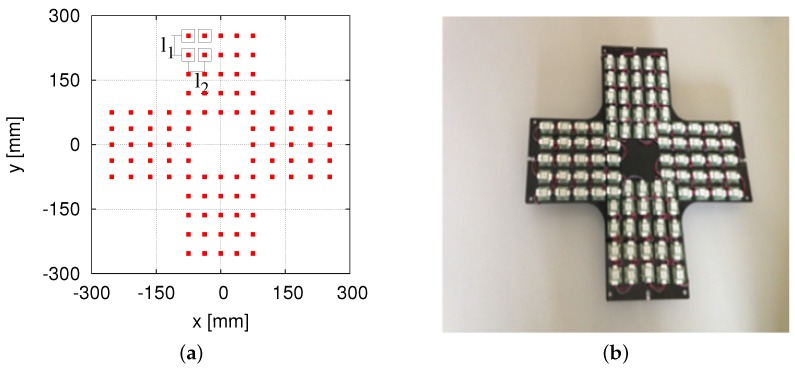
The 96 HF-band RFID readers. (**a**) Arrangement; (**b**) installation.

As shown in Figure 4a, the 96 small-sized HF-band RFID readers are arranged in a cross pattern. The RFID reader is small, with the size of one reader antenna being 30 × 30 mm${}^{2}$. Our RFID reader antenna is much smaller than the large antenna (660 × 300 mm${}^{2}$) employed in a previous work [24]. In this system, $l_{1}$ = 44.5 mm and $l_{2}$ = 37.5 mm. Figure 4b shows the 96 RFID readers designed and developed by us previously [16]. The RFID system makes the self-localization stable and accurate. However, the production cost is too high for a service robot being applied to public facilities.

**Figure 5:**
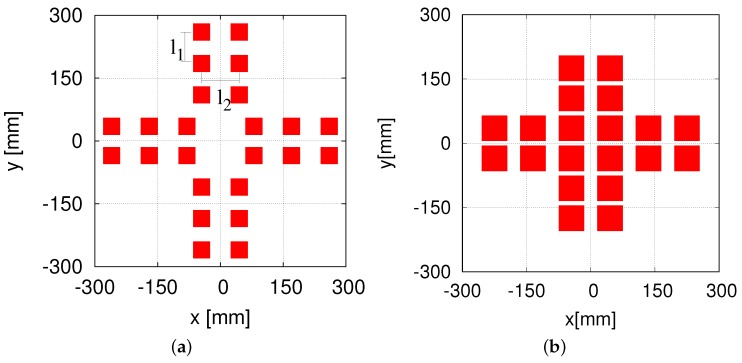
Arrangements of the 24 and 20 RFID readers. (**a**) the 24 readers; (**b**) the 20 readers.

To cut down the production cost while maintaining the high accuracy of the self-localization, we attempted to configure a low-cost HF-band RFID system by increasing the antenna size appropriately and reducing the number of RFID readers. We first reduced the number of readers to 24 (Figure 5a), which is a quarter of the original 96 RFID readers. This is because we increased the size of one antenna to 60 × 60 mm${}^{2}$, which is four-times the antenna size in the case of using 96 RFID readers. The 24-RFID-reader system performed well; further details of this system can be found elsewhere [29]. Then, we made a slight adjustment by reducing the number of readers to 20, as shown in Figure 5b. Specifically, we reduced the number of readers by four and instead placed four readers at the center in order to test the system. Further details have been reported elsewhere [30]. Both the 24-RFID-reader and the 20-RFID-reader systems were able to locate the robot accurately and stably. We wished to continue reducing the number of readers to six, i.e., a quarter of the 24 RFID readers. However, due to the robot, the readers have to be arranged in a cross pattern. Given the difficulty in arranging six readers in a cross pattern, we made a slight adjustment and used eight RFID readers instead.

### 4.2. System Using Eight HF-Band RFID Readers

**Figure 6:**
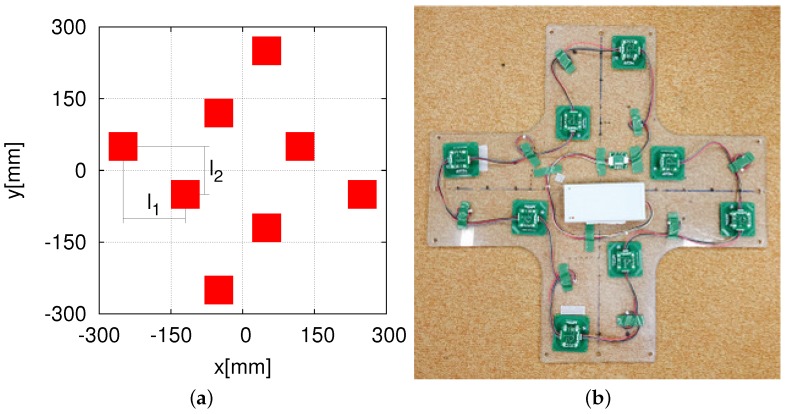
A new scheme for eight RFID readers. (**a**) Arrangement; (**b**) installation.

Figure 6 shows the newly-designed and built eight-RFID-reader system. The size of one RFID reader antenna in this system is 60 × 60 mm${}^{2}$, which is the same as the size of the antenna in the 24-RFID-reader system. For this new system, the intervals shown in Figure 6 are as follows: $l_{1}$ = 100 mm in the *x* direction and $l_{2}$ = 100 mm in the *y* direction.

### 4.3. Configurations of RFID Tags with Different Densities

**Figure 7:**
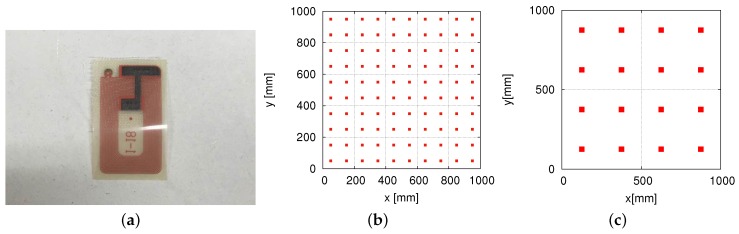
RFID tags in a lattice pattern. (**a**) Passive RFID tag; (**b**) 100 tags/m${}^{2}$; (**c**) 16 tags/m${}^{2}$.

The production cost depends on not only the configuration of the readers, but also the configuration of the tags embedded in the robot’s environment. A configuration with fewer tags is less expensive. Figure 7a shows a small passive RFID tag used by us. The size of the passive RFID tag is 10 × 20 mm${}^{2}$. As shown in Figure 7, the RFID tags are arranged in a lattice pattern. We investigated the performances of RFID systems in the case of using RFID tags arranged in a lattice pattern with different densities: 400 tags/m${}^{2}$, 100 tags/m${}^{2}$, 25 tags/m${}^{2}$ and 16 tags/m${}^{2}$. Figure 7b,c shows the configurations of RFID tags with densities of 100 tags/m${}^{2}$ and 16 tags/m${}^{2}$, respectively.

## 5. Likelihood Models for Tag Detection

### 5.1. RFID Tag Detection Model

**Figure 8:**
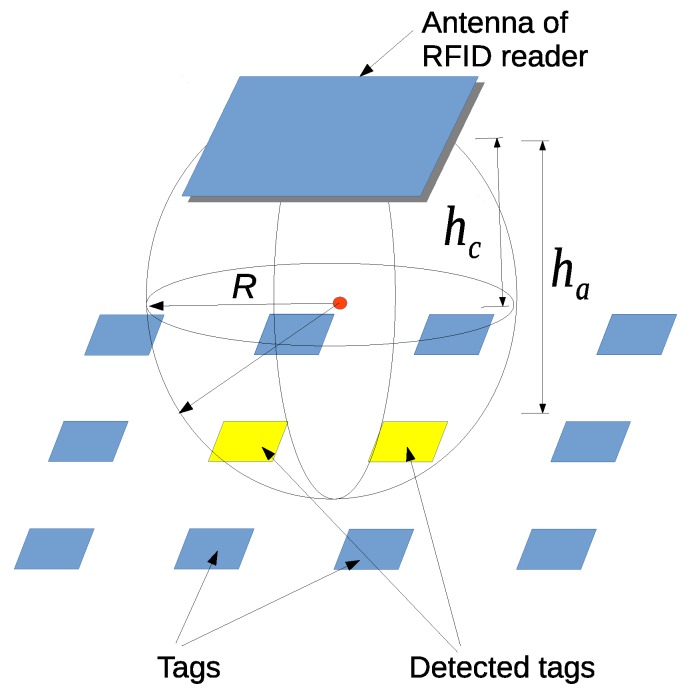
ID tag detection model for a small-sized RFID reader antenna.

An HF-band RFID reader detects a tag reliably if the tag is in the detection range. We model the detection range as follows. Figure 8 shows the model for ID tag detection. In this figure, the detection area is represented by the sphere drawn using the solid black line. Specifically, this sphere represents the detection range of one RFID reader. The radius of the detection range is denoted by *R*. The red dot represents the center of the detection range, which is just below the RFID reader antenna at a distance of $h_{c}$. The height of one RFID reader antenna is given as ${}^{w}z_{r_{i}}$ = $h_{a}$. Tags are embedded in the carpet on the floor. A tag is detected if and only if it is within the detection range of one RFID reader antenna, which is illustrated in Figure 8 and expressed in Equation (5). In the simulation part, we use this tag detection model to simulate the RFID reader.

(5) $${{(}^{w}x_{r_{i}}{-}^{w}x_{tag_{j}})}^{2}+{{(}^{w}y_{r_{i}}{-}^{w}y_{tag_{j}})}^{2}+{{(}^{w}z_{r_{i}}-h_{c}{-}^{w}z_{tag_{j}})}^{2}<R^{2}$$

**Figure 9:**
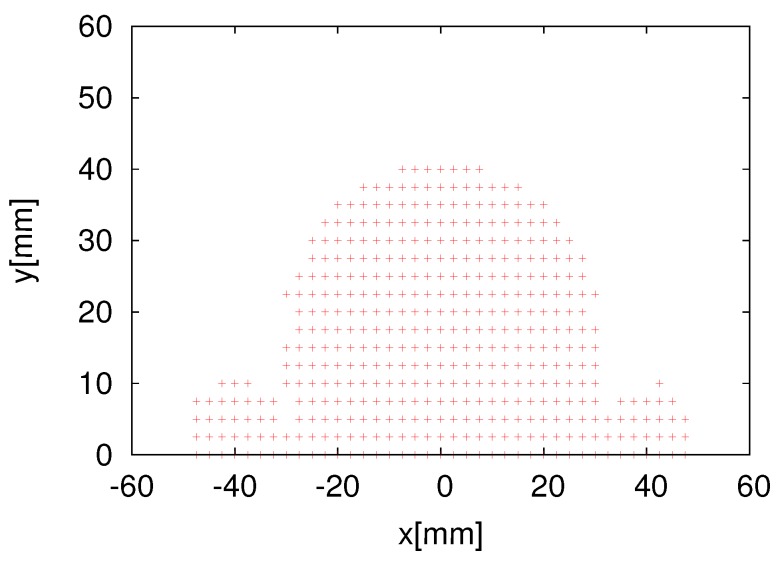
Detection area of the RFID reader antenna (data provided by Art Finex Co., Ltd., 6-1-33, Kamikoubata, Sabae, Fukui, Japan).

**Figure 10:**
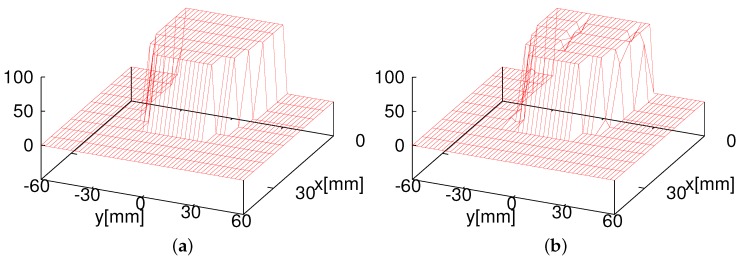
Tag detection rate at different heights (data provided by Art Finex Co., Ltd.). (**a**) 15 mm; (**b**) 20 mm.

Figure 9 shows the tag detection area of one RFID reader antenna whose size is 60 × 60 mm${}^{2}$. Figure 10 shows the cross-sectional views of the tag detection area and the success rates at heights of 15 mm and 20 mm. The *z*-axis represents the tag detection rate when the tag is located at (x,y). Use of a likelihood function for the measurement model is crucial for ensuring the accuracy of self-localization. Conventional studies employed a Gaussian distribution as the likelihood function. Figure 10 illustrates that the detection rate is almost 100% if the tag is in the detection range and almost 0% if the tag is outside the range. This suggests that a Gaussian distribution is unsuitable for the RFID system. Therefore, we investigated two likelihood functions: a Gaussian distribution and a combination of the Gaussian distribution and step function.

### 5.2. Two Different Likelihood Models

**Figure 11:**
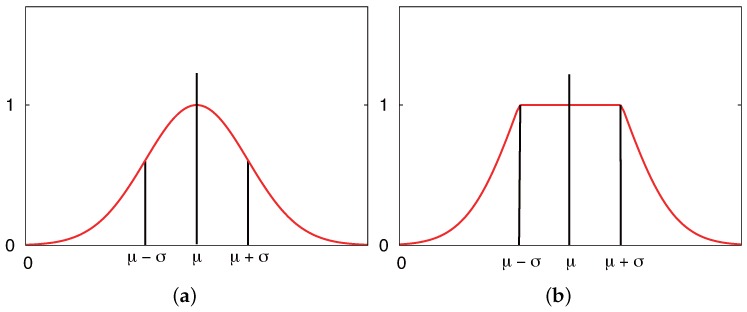
Likelihood functions for the measurement model of tag detection. (**a**) Gaussian model; (**b**) combination model.

We establish two different likelihood functions for the measurement models of MCL, as shown in Figure 11. One likelihood function is defined by the distance between a reader and the ID tag detected by it. This likelihood function often uses the Gaussian model, as shown in Figure 11a. Particles tend to gather around the center of the Gaussian distribution because the weights of the particles are calculated by the Gaussian distribution function N(μ,σ), and the closer a particle to *μ*, the higher is its assigned weight. Figure 11b shows a combination of the Gaussian distribution and the stepwise function, hereafter referred to as the combination model, newly designed in this study. The combination likelihood function is defined as given in Equation (6):

(6) $$p\left(tag_{j},r_{i}\right)=\left\{\begin{array}{c} {1\mathrm{if}||}^{w}x_{tag_{j}}{-}^{w}x_{r_{i}}\left|\right|<\sigma \\ \beta \exp\left(-\frac{1}{2\sigma^{2}}{{(}^{w}x_{tag_{j}}{-}^{w}x_{r_{i}})}^{T}{(}^{w}x_{tag_{j}}{-}^{w}x_{r_{i}})\right)\mathrm{else} \end{array}\right.$$

where *β* is a constant. The weight of the particles in the range defined between the center and *σ* is one. Otherwise, the weight of the particles reduces according to the distance between the reader and the tag. We evaluate these two different likelihood function models in both a simulation and a real environment.

### 5.3. Simulations of Self-Localization by a 96 HF-Band RFID Reader System Using Two Likelihood Models

In the simulations of the 96 HF-band RFID reader system, the height $h_{a}$ of the antenna is set to 20 mm, and the detection radius *R* is 15 mm. The center of the detection area of a reader antenna is just below the antenna at a distance $h_{c}$ of 8 mm. Table 2 presents the simulation results obtained using the 96 HF-band RFID readers with the two different likelihood models. The RFID tags are arranged in a lattice pattern with four different densities, as described in Section 4.3.

The results in the table illustrate that the 96 HF-band RFID reader system performs highly accurate self-localization with average errors of less than 5 mm in both the *x* and *y* directions, irrespective of whether the Gaussian model or the combination model is used. The variances and maximum errors listed in Table 2 also demonstrate that the proposed system is quite stable. Though the data in the table show that both the likelihood models provide almost the same accuracy of self-localization, the average errors in the case of using the combination model are slightly smaller than those in the case of using the Gaussian model. This result supports the hypothesis that the combination model works better than the Gaussian model. In general, the self-localization accuracy decreases when the tag density decreases, and from the data in Table 2, it is seen that the density of 400 tags/m${}^{2}$ provides the best accuracy. Further, the density of 100 tags/m${}^{2}$ is better than densities of 25 tags/m${}^{2}$ and 16 tags/m${}^{2}$. However, Table 2 reveals that the average error for the density of 16 tags/m${}^{2}$ is slightly smaller than that for the density of 25 tags/m${}^{2}$. The self-localization performance is easily affected by the configurations of the readers and tags and the likelihood models. Additionally, the routes of the robot also affect the accuracy, especially under conditions of using low densities of tags, such as 25 tags/m${}^{2}$ and 16 tags/m${}^{2}$, because the self-localization is based on the reading of the tags’ information by the RFID readers. This is the reason why the average errors in the case of 16 tags/m${}^{2}$ are slightly smaller than those in the case of 25 tags/m${}^{2}$.

Table 3 presents the simulation results for the 96 HF-band RFID reader system without the particle reinitialization. The simulations were performed with a tag density of 100 tags/m${}^{2}$ and using the two likelihood models. The average errors, maximum errors and variances in this simulation are much larger than the values listed in Table 2. This is because the particles would be reinitialized under the condition that their weight is too small for the system to estimate the position of the robot. Unnecessary particles also would be eliminated to make the system locate the robot quickly and accurately. This proves that our proposed particle reinitialization method enables more accurate and stable self-localization.

### 5.4. Simulations of Self-Localization by the Eight HF-Band RFID Reader System Using Two Different Likelihood Models

In the simulation with the eight HF-band RFID reader system, *R* = 30 mm, $h_{c}$ = 24 mm and ${}^{w}z_{r_{i}}$ = 24 mm. Table 4 lists the simulation results in the case of using the eight HF-band RFID reader system. The average errors in the simulation are less than 10 mm in both the *x* and *y* directions for both the Gaussian and the combination models. As was the case with the self-localization using the 96 HF-band RFID reader system, the self-localization using the eight HF-band RFID reader system was also highly accurate and stable, despite the reduction in the number of readers from 96 to eight. Furthermore, as was the case with the simulation using the 96 HF-band RFID reader system, in this case, as well, the density of 400 tags/m${}^{2}$ provided the best self-localization accuracy. Further, the density of 100 tags/m${}^{2}$ provided better accuracy than did the densities of 25 tags/m${}^{2}$ and 16 tags/m${}^{2}$. The simulation results show that the average self-localization errors when using the combination model are smaller than those when using the Gaussian model under all of the tag arrangement conditions. This also proves our hypothesis that the combination model performs better self-localization than does the Gaussian model. It is found from Table 4 that the average errors for 25 tags/m${}^{2}$ are slightly larger than those for 16 tags/m${}^{2}$, which is the same as the result seen in Table 2. As described in Section 5.3, this result can be attributed to the routes of the robot selected by us.

## 6. Experiments in a Real Environment

**Figure 12:**
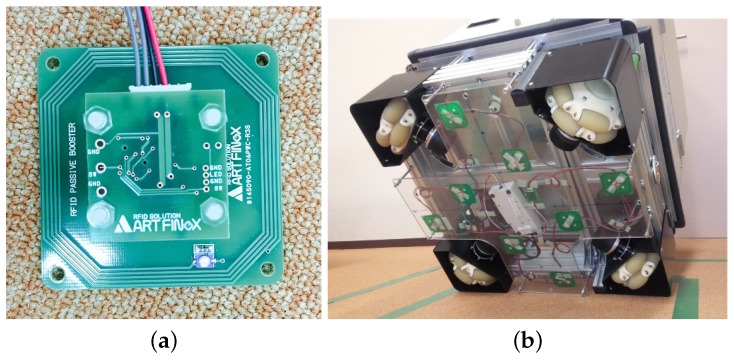
Installation of eight HF-band RFID readers at the bottom of an omni-directional vehicle. (**a**) An RFID reader; (**b**) bottom view.

The eight RFID reader system (Figure 6) was attached at the bottom of our omnidirectional vehicle, as shown in Figure 12. As mentioned earlier, Figure 9 shows the tag detection area of one RFID reader antenna of the eight HF-band RFID reader system, and Figure 10 shows the cross-sectional view of the tag detection area and the success rate at heights of 15 mm and 20 mm. Figure 10 demonstrates that the combination likelihood model is more suitable than the Gaussian likelihood model at the heights of 15 mm and 20 mm. This also proves our hypothesis in simulations that the combination model performs better than the Gaussian model. Next, we verified whether or not the combination model performs better than the Gaussian model in a real environment.

**Figure 13:**
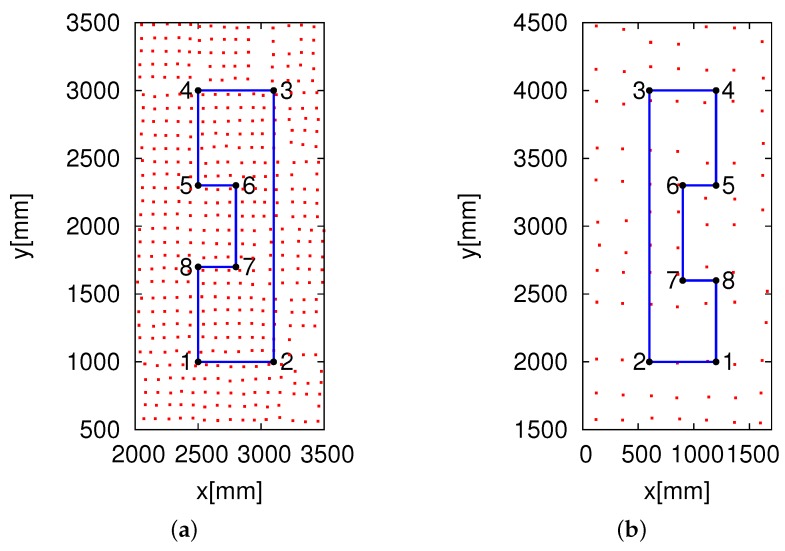
Experimental environment: (**a**) the installation of ID tags with a density of 100 tags/m${}^{2}$ and (**b**) the installation of ID tags with a density of 16 tags/m${}^{2}$. The red dots represent the ID tags, and the blue lines represent the experimental routes. The black dots represent statistic points.

As shown in Figure 13a, we verified the eight HF-band RFID reader system using the two different likelihood models in the real environment. The height of the readers was set as 15 mm because the reader antenna has a better tag detection success rate.

The speed of the robot was set as 100 mm/s. The eight RFID readers and the 96 RFID readers detected ID tags every 50 ms. The frequency of the eight RFID readers and 96 RFID readers was the same, i.e., 13.56 MHz. ID tags were embedded in the carpet on the floor with densities of 100 tags/m${}^{2}$ and 16 tags/m${}^{2}$ in the lattice pattern. We made the robot run the path shown in Figure 13, from Point 1 to Point 8. It was difficult to determine the localization error for every position along which the robot moved. To calculate the localization errors, we marked eight points at which the positions were already known, as shown in Figure 13. The robot stayed for 20 s at each of the eight points to perform self-localization, so that it could collect enough data to calculate the self-localization errors. We first verified our new eight HF-band RFID reader system. We also performed a comparison experiment with the 96 HF-band RFID reader system. To analyze the self-localization errors, we acquired a large amount of data through calculations at the eight points shown in Figure 13.

The experimental results for the 96 HF-band RFID reader system are presented in Table 5. At the density of 100 tags/m${}^{2}$, the average self-localization errors are smaller than 15 mm in the *x* direction and smaller than 25 mm in the *y* direction for both likelihood function models. The comparison of these two likelihood models reveals that in the *x* direction, the average self-localization error when using the combination model is 8.2 mm, which is smaller than that when using the Gaussian model. In the *y* direction, both the likelihood models perform self-localization with almost the same accuracy, with errors of 22.3 mm and 23.8 mm for the Gaussian and combination models, respectively. At the density of 16 tags/m${}^{2}$, the combination model performs better than the Gaussian model, where, as shown in Table 5, the average self-localization errors are 26.2 mm in the *x* direction and 18.0 mm in the *y* direction for the Gaussian model and 17.7 mm in the *x* direction and 13.5 mm in the *y* direction for the combination model. From these average self-localization errors, it can be said that the self-localization at the density of 16 tags/m${}^{2}$ is accurate enough: in the *x* direction, the errors at this density are only slightly larger than those at 100 tags/m${}^{2}$, and in the *y* direction, the errors at this density are smaller than those at 100 tags/m${}^{2}$. However, the self-localization at this density was not stable, as the maximum errors and variance were very large, as seen in Table 5. This is because the system could not detect tags at some places when the density of the ID tags became as low as 16 tags/m${}^{2}$. The detection area of one RFID reader is narrow so that the size of the 96 RFID reader antenna is small. Eventually, the self-localization errors increase correspondingly. With the configuration of 96 small RFID readers and the ID tag density of 16 tags/m${}^{2}$, the system is unable to locate the robot stably.

Table 6 presents the results of self-localization errors in the case of the eight HF-band RFID reader system. The results show that the eight HF-band RFID reader system with large antennas performs highly accurate self-localization. As is seen from the table, the average self-localization errors in the case of both of the likelihood models at the density of 100 tags/m${}^{2}$ are almost the same. However, as can be seen from the table, even though both of the likelihood models perform well at the density of 16 tags/m${}^{2}$, the combination model performs better than the Gaussian model. The maximum errors and variance at the density of 16 tags/m${}^{2}$ are much larger than those at 100 tags/m${}^{2}$; however, the system still maintains stable localization.

From Table 5 and Table 6, it can be seen that at the density of 100 tags/m${}^{2}$, the average errors in the *x* direction are much smaller than those in the *y* direction. At the density of 16 tags/m${}^{2}$, the average errors in the *x* direction are larger than those in the *y* direction. As the arrangement of the RFID reader antennas is the same in both the *x* and *y* directions, we consider that these differences in average errors were caused by the ID tag installation. Because it is difficult to ensure the installation of every two adjacent tags at the same interval, the installation errors cannot be prevented.

From a comparison of the two RFID systems, we found that both systems performed highly accurate self-localization at the density of 100 tags/m${}^{2}$ and that there was only a slight difference in the average self-localization errors when the combination model was used. The eight HF-band RFID reader system performed slightly better than the 96 HF-band RFID reader system. The difference between the two RFID systems was that the eight HF-band RFID reader system could locate the robot accurately and stably under the condition of using a low density of ID tags, 16 tags/m${}^{2}$, whereas the system with the 96 small RFID readers could not. This is because the eight RFID readers are equipped with enlarged antennas in order to widen the tag detection range of a single RFID reader. The efficient RFID readers of the two RFID systems that detect ID tags are almost the same. Moreover, we used MCL for self-localization, which could enable the robot to be located precisely even if only one or two tags were detected. The experimental results demonstrate the efficiency of our newly-developed eight HF-band RFID reader system with large antennas. This system performs robot self-localization stably and accurately, which proves that eight RFID readers with large antennas, instead of 96 RFID readers, can provide sufficient self-localization accuracy for robot localization. Moreover, because we use eight RFID readers, the production cost of the system can be reduced significantly in comparison to that of the 96 HF-band RFID reader system.

### Trajectory of Real-Time Self-Localization

**Figure 14:**
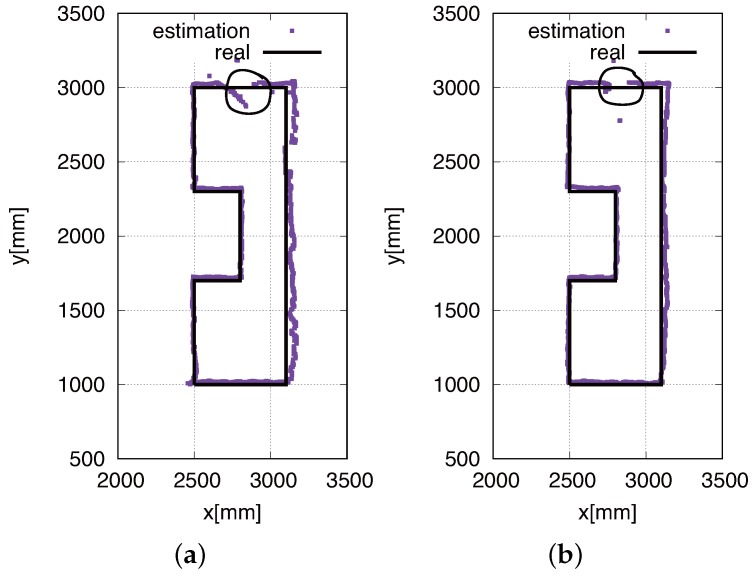
Real-time self-localization trajectory obtained using (**a**) the eight HF-band RFID reader system using the conventional Gaussian model and (**b**) the eight HF-band RFID reader system using the proposed likelihood model. The density of the ID tags was 100 tags/m${}^{2}$, and the robot speed was 100 mm/s.

To verify the real-time self-localization performance of the developed eight HF-band RFID reader system, we obtained the statistics of the real-time localization of the robot. Figure 14 shows the trajectories of our proposed eight HF-band RFID reader system. Figure 14a shows the real-time self-localization trajectory obtained using the conventional Gaussian model, whereas Figure 14b shows that obtained using our proposed likelihood model. The comparison of Figure 14a,b reveals that the proposed likelihood model works better than the conventional method employing the Gaussian model. As seen in both figures, Figure 14a,b, the robot could not perform high-accuracy localization in the area indicated by the circle. This is because the system could not detect ID tags, since they had an installation error. On the other side, our eight RFID reader antenna is small; once tags are distributed in the gap of the detection area, the system could not detect tags. The reader detection area is shown in Figure 9, and the installed RFID tags are shown in Figure 13.

**Figure 15:**
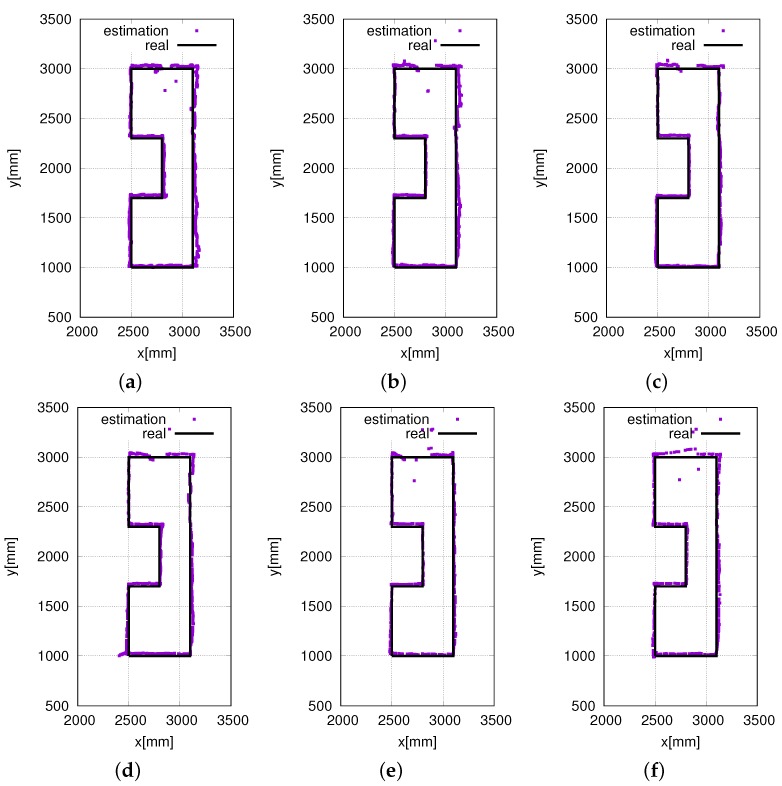
Real-time self-localization trajectories at different speeds. (**a**) 50 mm/s; (**b**) 150 mm/s; (**c**) 200 mm/s; (**d**) 250 mm/s; (**e**) 300 mm/s; (**f**) 350 mm/s.

In our experiments, we also validated the performance of the proposed eight HF-band RFID reader system at different speeds. Figure 14b shows the real-time localization at a speed of 100 mm/s. Figure 15 shows the real-time localization at speeds in the range of 50 mm/s to 350 mm/s at intervals of 50 mm/s. From Figure 15a to Figure 15f, it is clear that the localization accuracy does not decrease with an increase in the speed. Furthermore, as shown in Figure 15, to a certain extent, the system even performs better at high speeds. This is because when the robot moves at low speeds, the noise of the motors has a much greater effect on the system performance. In contrast, when the robot moves at high speeds, the noise of the motors has less time to have an effect on the system performance. Above all, the proposed RFID system was able to realize highly accurate and stable localization at various speeds. This proves the efficiency and stability of the proposed RFID system.

## 7. Conclusions

In this study, we achieved stable and accurate self-localization of an indoor mobile robot by using a newly-developed RFID system and investigated the self-localization performance at different configurations of the RFID system. We eventually determined a novel configuration of the RFID system that has low production cost and provides highly accurate and stable self-localization. In order to make the self-localization realized by the proposed RFID system more accurate and efficient, we applied an efficient particle reinitialization method to MCL. We designed two different likelihood models, a Gaussian model and a combination model (a combination of the Gaussian distribution and the step function), to investigate their influence on the self-localization performance. We verified both of the likelihood models experimentally in a real environment. The experimental results proved our hypothesis that the combination model performs better than the Gaussian model. Results of both simulations and real environment experiments demonstrate that the proposed configuration consisting of eight HF-band RFID readers provides sufficiently high accuracy of self-localization.

## Figures and Tables

**Table 1:** Related works: self-Localization.

Works	Band	Reader Number	Arrangement of Tags	Sensors	Method	Localization Error
Miguel Pinto [18]	×	×	×	omni-camera, LRF	based on Kalman filter	<80 mm
Dirk Hahnel [8]	UHF	2	attached to objects	LRF	Monte Carlo localization	much better than only using LRF
Dany Fortin-Simard [9]	UHF	4	attached to objects	different sensors and effectors	filters using an elliptical model	141.2 mm
Lei Yang [19]	UHF	1	uniformly distributed	×	hybrid particle filter	186 mm
Kodaka [12]	LF	2	lattice pattern, 16 tags/m${}^{2}$	×	particle filter	<100 mm
Sunhong Park [13]	HF	1	grid-like pattern, 9 tags/m${}^{2}$	×	trigonometric functions	133 mm in *x*-axis and 57 mm in *y*-axis
Soonshin Han [14]	HF	1	triangular pattern, 400 tags/m${}^{2}$	×	based on motion-continuity property	16 mm
Takahashi [15]	HF	8	lattice, 100 tags/m${}^{2}$	×	kinematics method	<17 mm
Takahashi [16]	HF	96	lattice, 100 tags/m${}^{2}$	×	Monte Carlo Localization	11.8 mm in*x*-axis and 18.6 mm in *y*-axis
Po Yang [24]	HF	1	sparsely distributed	×	a simple and efficient localization method	36 mm in *x*-axis and 38 mm in *y*-axis
Po Yang [25]	HF	1	dense passive RFID tag distribution, 100 tags/m${}^{2}$	×	particle filter	27 mm in *x*-axis and 46 mm in *y*-axis
Mohd Yazed Ahmad [26]	HF	1	sparsely distributed	×	using tag information and wheel encoder data	40.5 mm

**Table 2:** Self-localization performances of the 96 HF-band RFID reader system using two likelihood models.

Tag Density (tags/m${}^{2}$)	Likelihood Model	Error by Mean			Error by Max			Variance		
		*x* (mm)	*y* (mm)	*θ* (rad)	*x* (mm)	*y* (mm)	*θ* (rad)	*x* (mm${}^{2}$)	*y* (mm${}^{2}$)	*θ* (rad${}^{2}$)
400	Gaussian	1.6	1.5	0.003	9.4	8.3	0.015	2.6	2.5	0.000
400	Combination	1.3	1.2	0.005	7.0	6.9	0.019	1.2	1.2	0.000
100	Gaussian	2.2	1.9	0.003	9.5	9.1	0.018	2.9	2.6	0.000
100	Combination	1.7	1.5	0.004	7.7	7.7	0.022	1.6	1.4	0.000
25	Gaussian	4.4	4.4	0.010	16.5	33.8	0.120	8.6	20.0	0.000
25	Combination	4.0	4.2	0.011	12.4	28.6	0.099	8.4	18.7	0.000
16	Gaussian	3.8	3.7	0.007	13.3	20.7	0.055	6.6	6.6	0.000
16	Combination	3.8	3.0	0.006	13.3	14.2	0.085	6.1	4.8	0.000

**Table 3:** Self-localization performances of the 96 HF-band RFID reader system without reinitialization.

Tag Density (tags/m${}^{2}$)	Likelihood Model	Error by Mean			Error by Max			Variance		
		*x* (mm)	*y* (mm)	*θ* (rad)	*x* (mm)	*y* (mm)	*θ* (rad)	*x* (mm${}^{2}$)	*y* mm${}^{2}$)	*θ* (rad${}^{2}$)
100	Gaussian	21.6	21.3	0.490	169.6	153.3	1.494	737.8	750.6	0.211
100	Combination	21.8	21.5	0.504	163.3	152.3	1.515	717.6	719.6	0.201

**Table 4:** Self-localization performances of the eight HF-band RFID reader system using two likelihood models.

Tag Density (tags/m${}^{2}$)	Likelihood Model	Error by Mean			Error by Max			Variance		
		*x* (mm)	*y* (mm)	*θ* (rad)	*x* (mm)	*y* (mm)	*θ* (rad)	*x* (mm${}^{2}$)	*y* (mm${}^{2}$)	*θ* (rad${}^{2}$)
400	Gaussian	3.1	2.2	0.007	13.1	8.9	0.027	5.2	2.5	0.000
400	Combination	2.5	2.2	0.010	12.2	10.7	0.038	3.4	2.8	0.000
100	Gaussian	5.2	3.1	0.012	22.3	13.9	0.127	16.7	5.0	0.000
100	Combination	4.4	3.1	0.014	18.3	16.1	0.086	14.3	6.8	0.000
25	Gaussian	7.4	5.9	0.022	23.5	28.3	0.075	23.1	21.7	0.000
25	Combination	6.7	5.3	0.020	39.7	32.7	0.089	33.1	22.8	0.000
16	Gaussian	6.7	5.0	0.015	24.8	21.9	0.055	26.0	14.3	0.000
16	Combination	6.2	4.9	0.019	31.3	50.1	0.120	22.9	23.8	0.000

**Table 5:** Self-localization errors with 96 HF-band RFID reader systems.

Density (tags/m${}^{2}$)	Likelihood Model	Error by Mean			Error by Max			Variance		
		*x* (mm)	*y* (mm)	*θ* (rad)	*x* (mm)	*y* (mm)	*θ* (rad)	*x* (mm${}^{2}$)	*y* (mm${}^{2}$)	*θ* (rad${}^{2}$)
100	Gaussian	14.2	22.3	0.030	37.5	44.4	0.068	98.7	128.8	0.000
100	Combination	8.2	23.8	0.032	30.4	43.0	0.084	59.0	93.1	0.000
16	Gaussian	26.2	18.0	0.107	1867.3	562.4	0.402	4331.3	601.2	0.009
16	Combination	17.7	13.5	0.061	2699.0	820.2	0.248	7075.5	731.1	0.002

**Table 6:** Self-localization errors with the eight HF-band RFID reader systems.

Density (tags/m${}^{2}$)	Likelihood Model	Error by Mean			Error by Max			Variance		
		*x* (mm)	*y* (mm)	*θ* (rad)	*x* (mm)	*y* (mm)	*θ* (rad)	*x* (mm${}^{2}$)	*y* (mm${}^{2}$)	*θ* (rad${}^{2}$)
100	Gaussian	5.7	24.3	0.020	18.1	40.0	0.053	13.2	39.2	0.000
100	Combination	4.7	22.8	0.019	16.8	38.3	0.075	11.3	52.1	0.000
16	Gaussian	33.3	12.9	0.056	88.3	40.0	0.105	502.8	84.0	0.001
16	Combination	13.0	8.7	0.046	94.0	42.5	0.097	249.2	57.9	0.001
